# Influence of racial crossing on testicular structure and spermatogenesis in sheep Santa Inês and crossbred Santa Inês and Dorper

**DOI:** 10.21451/1984-3143-AR2017-0013

**Published:** 2018-08-16

**Authors:** Morgana Santos Araújo, Isac Gabriel Cunha dos Santos, Jean Rodrigues Carvalho, Juanna D’Arc Fonseca Santos, Antônio Francisco da Silva Lisboa, Amilton Cesar dos Santos, Manoel Lopes da Silva, Feliciana Clara Fonseca Machado, Antonio Augusto Nascimento Machado

**Affiliations:** 1 Universidade Federal Piauí, Bom Jesus, PI, 64900-000, Brazil.; 2 Programa de Pós-graduação em Anatomia dos Animais Domésticos e Silvestres, Faculdade de Medicina Veterinária e Zootecnia, São Paulo, SP, 05508-010, Brazil.; 3 Departamento de Medicina Veterinária, Universidade Federal do Piauí, Bom Jesus, PI, 64900-000, Brazil.

**Keywords:** **:** sheep, spermatogenic efficiency, testicles

## Abstract

The study aimed at evaluating the testicular structure and spermatogenesis of Santa Inês and Santa Inês and Dorper crossbred sheep. Sixteen testicles of the animals under study were used, in order to evaluate the volumetric proportion, the diameter of the tubules, the height of seminiferous epithelium, the frequency of stages of the seminiferous epithelium cycle and the spermatogenic yield. The data were submitted to Student-Newman-Keulstestat 5% significance. The tubular diameter was 173.12 ± 29.09 μm and 185.71 ± 29.7 μm, and the height of the seminiferous epithelium was 52.29 ± 9.98 μm and 56.68 ± 11.25 μm, for the crossbred and the Santa Inês, respectively. There was no difference between the testicular compartments and the frequency of the stages of the seminiferous epithelium cycle between the groups. Santa Inês sheep presented a number of 15.36 ± 4.49 spermatocytes in the pre-leptotene/leptotene stage and 27.42 ± 6.65 spermatocytes in the pachytene, whereas the crossbred presented 13.18 ± 5.19 and 23.48 ± 7.80, respectively. The crossbred showed higher meiotic yield (3.98 ± 1.28) and spermatogenesis yield (3.71 ± 1.02). It is concluded there are differences in testicular morphology and spermatogenesis between Santa Inês sheep and crossbred Dorper/Santa Inês, indicating that the crossbreeding between the Santa Inês and Dorper animals allows a gain in the reproductive potential.

## Introduction

The knowledge of the reproductive function has been pointed as a fundamental factor for the establishment of adequate management programs ( Nunes *et al.,* 2013 ). Therefore, it is essential to evaluate the reproductive capacity of the males, in order to obtain animals capable of transmitting characteristics that will improve the productive system ( Pacheco and Quirino, 2010 ).

Sperm formation (spermatogenesis) involves cytological processes, such as proliferation, differentiation and cell transformation, as well as the development of progenitor cells in the seminiferous tubules. When studying the spermatogenic process, both classifications for the stages of the cycle of seminiferous epithelium and the quantification of the cell types should be considered, in order to verify their development throughout the cycle ( Almeida *et al.,* 2000 ; Assis Neto *et al.,* 2003). This quantification makes it possible to elucidate the way in which these cells normally undergo through division and renewal, allowing the determination of the coefficient efficiency of spermatogenesis ( Castro *et al.,* 1997 ).

The yield of the spermatogenic process, obtained through the numerical ratios of the spermatogenic cells and through the sections of seminiferous tubule, allows the evaluation of the productive capacity of spermatozoa. This variation is considered a fundamental variable for the determination of males destined for reproduction, in order to know in which cellular phases occur the losses, conceding the estimation of the percentage of these cells and the comparison between the species (Assis Neto *et al.,* 2003; Costa *et al.,* 2004 ; Nunes *et al.,* 2013 ).

The evaluation of the spermatogenic activity between distinct racial groups within the same species becomes an instrument of great importance, which grants the knowledge of the reproductive potential of a certain blood group, thus contributing to the selection of quality reproducers. No studies were found comparing spermatogenesis among sheep breeds, a fact that motivated this research. There are studies such as Maia *et al*. (2011) , which trace a comparative study of the quality of semen in sheep, but without addressing the morphological aspects. In cattle, however, Andreussi *et al.* (2014) developed this type of comparative study, and they found differences when evaluating the morphology of testis and spermatogenic yield between five Zebu cattle breeds used in Brazil (Nelore, Polled Nelore, Gir, Guzerá, and Tabapuã).

In this aspect, considering the relevance in the scope of the reproduction of studies involving the reproductive morphophysiology, and in the search for animals that are more productive, with better reproductive characteristics, the aim of this study was to compare testicular structure and spermatogenesis of Santa Inês and crossbred Santa Inês/Dorper sheep.

## Material and Methods

### Animals and experimental conditions

The research was authorized by the Committee for Ethics and Animal Experimentation of the Federal University of Piauí, under N^o^. 098/15. Sixteen testicles were used, from eight animals, Santa Ines and crossbred-Santa Ines x Dorper. A general clinical examination of the reproductive system was performed, in order to verify the integrity of these organs through palpation. The animals were healthy, with adequate body condition and ages between 18 and 24 months.

The analysis was conducted in the experimental sheepfold of Universidade Federal do Piauí, Campus Professor Cinobelina Elvas at Bom Jesus City, located in the state of Piauí, Brazil. This town is located at 09^o^04’28” of South latitude, 44^o^21’31” of West longitude and 277 m of altitude. During the experimental period, between May and July 2015, the minimum and maximum temperature and relative humidity of the air corresponded on average to 19.7ºC, 33.9ºC and 46.6% respectively, according to data from the National Institute of Meteorology ( Instituto Nacional de Meteorologia - INMET, 2016 ). The animals were subjected to a 60-day adaptation period prior to the experiments. The feeding consisted of grass (*Penisetun purpureum*) offered at will, proper commercial supplementation, mineral salt and water *ad libitum*. Feeding was offered in the morning and late afternoon.

### Procedure for analysis in light microscopy

Before castration, the animals were weighed. After castration the testicles were weighed for the calculation of the Gonadosomatic Index (GI), which is obtained by the weight of the testicle divided by the body weight. Then, the testicles were sectioned and the fragments were fixed in Bouin's solution and refrigerated at 8°C for 24 h. The fragments were dehydrated in increasing alcohol solutions (from 70 to 100%). Then, they were diaphanized in Xylol for later inclusion in paraffin blocks. Sections of 4 μm were obtained on a microtome and stained with Hematoxylin-Eosin. The slides were analyzed under light microscope.

### Obtaining volumetric proportion

The volumetric proportions of the compartments of the testicles were obtained by means of the photomicrograph evaluation of 20 sequential fields per blade, analyzing all parts of it uniformly, using a reticle containing 441 intersections ( Elias *et al.,* 1971 ). The points that coincided in the regions of the testicular compartments were counted, totaling 8820 points, in which the tubular compartment was considered: the *tunica propria *, the seminiferous epithelium, the tubular light, and the elements of the interstitial compartment of the tubules: Leydig cells, testicular vessels and connective tissue, with magnification of 400X.

### Frequency of stages of the cycle of the seminiferous epithelium (CES):

The frequency of CES stages of sheep was determined through the tubular morphology method ( Berndtson, 1977 ; Ortavant *et al.,* 1977 ), by observing 500 transverse sections (at 400X magnification) of the seminiferous tubules from each animal, followed by a distance of at least 500μm between two sections evaluated, in order to prevent tubular sections of the same segment. The following formula was used to obtain the frequencies:

$$Frequency\;=\;\frac{Frequency\;of\;each\;stage\;observed}{Total\;observations}\;x\;100$$

### Tubular diameter and height of the seminiferous epithelium

For the seminiferous tubule diameter measurements and the seminiferous epithelium height, 60 transverse sections of seminiferous tubules in stage 1 of the cycle were analyzed (400X magnification), choosing tubules with rounded contour.

### Population of spermatogenic cells:

The population of cells present in Stage 1 of the CES was quantified through the analysis of 20 transverse sections of seminiferous tubules (400X magnification), with rounded contour.. Gross count of spermatogenic cells was corrected by nuclear/nucleolar diameter and the thickness of the histological section, according to the formula proposed by Abercrombie (1946) , modified by Amann and Almquist (1962) :

$$Corrected\;number\;=\;obtained\;count\;x\frac{Cutting\;thickness}{\text{Cutting thickness}+\sqrt{{\frac{\text{(DM)}}{\text{2}}}^{\text{2}}\text{-}{\frac{\text{(DM)}}{\text{4}}}^{\text{2}}}}$$

By means of the measurements of ten germ cell nuclei and Sertoli cells per animal, the Mean Nuclear Diameter was obtained (100X magnification).

### Spermatogenic yield

Spermatogenesis yield was determined by estimating the following cell types at stage 1 of the seminiferous epithelium cycle:

1. Yield or coefficient of efficiency of mitoses: calculated for the ratio between primary spermatocytes in pre-leptotene and spermatogonia;
2. Meiotic yield: calculated by the ratio between rounded spermatid and primary spermatocytes in pachytene;
3. General spermatogenesis yield: calculated by the ratio of round spermatids and spermatogonia;
4. Sertoli cell efficiency: calculated by the ratio of the total number of rounded spermatids to the total number of Sertoli cells.

### Statistical analysis

The statistical analysis was performed using ASISTAT software. The data were first submitted to the Shapiro-Wilks test to evaluate the normality of distribution. Posteriorly, analysis of variance (ANOVA) was performed for a completely randomized design, with two treatments and four replications, followed by Student-Newman-Keuls (SNK) test at a 5% level of significance, to compare the means.

## Results

Table 1 presents the results obtained from the data on biometric parameters and testicular morphometry among the studied groups. It is possible to verify that no statistical difference was found between the group’s body weight, testicular weight, Gonadosomatic Index and for the volume of testicular compartments. However, there was statistical difference in the measurements of the seminiferous tubule diameter and in the height of the seminiferous epithelium among the groups (P < 0.05).

**Table 1:** Mean ± standard deviation of testicular biometric parameters of Santa Inês and crossbred Santa Inês/Dorper (SI/DO).

	Crossbred SI/DO	Santa Inês
Body Weight (Kg)	50.25 ± 2.06^a^	48.50 ± 9.88^a^
Testicular Weight (g)	130.86 ± 39.60^a^	142.83 ± 60.41^a^
Gonadossomatic Index (%)	0.26 ±0.08^a^	0.28 ± 0.07^a^
Volume of testicular compartments (%)		
Tubular	77.02 ± 17.58^a^	76.61 ± 18.74^a^
Lamina propria	10.95 ± 5.33^a^	10.90 ± 5.48^a^
Seminiferous epithelium	53.65 ± 13.96^a^	54.62 ± 14.05^a^
Lumen	17.96 ± 10.12^a^	16.86 ± 9.44^a^
Interstitial	25.88 ± 20.47^a^	25.94 ± 21.08^a^
Leydig Cells	4.61 ± 2.8^a^	4.61 ± 2.7^a^
Connective Tissue	24.16 ± 18.97^a^	23.49 ± 19.17^a^
Testicular Vessels	4.98 ± 3.96^a^	4.97 ± 4.33^a^
Tubular Diameter (µm)	173.12 ± 29.09^b^	185.71 ± 29.73^a^
Height of the seminiferous epithelium (µm)	52.29 ± 9.98^b^	56.68 ±11.25^a^

* Different letters in the same line expressed significant differences (P <0.05) between Santa Inês and crossbred SI/DO for the Student-Newman-Keuls test (SNK).

The frequencies of the stages of the seminiferous epithelium cycle in the studied groups are distributed in Table 2 . Regarding this parameter, there was no significant difference among the groups.

**Table 2:** Mean ± standard deviation of the relative frequencies of the stages that compose the cycle of seminiferous epithelium in Santa Inês and crossbred Santa Inês/Dorper (SI/DO).

Stages	Crossbred SI-DO	Santa Inês	Phases of spermatogenesis
1	21.65 ± 3.71^a^	20.05 ± 3.34^a^	
2	13.15 ± 1.99^a^	13.80 ± 5.55^a^	Pre-meiotic
3	15.90 ± 3.68^a^	13.80 ± 0.43^a^	
4	7.05 ± 2.54^a^	7.25 ± 0.44^a^	Meiotic
5	10.65 ± 2.18^a^	11.45 ± 4.87^a^	
6	11.35 ± 2.40^a^	10.85 ± 1.79^a^	Post-meiotic
7	7.55 ± 2.39^a^	8.00 ± 1.55^a^	
8	12.90 ± 1.7^a^	14.80 ± 4.82^a^	

* Different letters in the same line expressed significant differences (P <0.05) between Santa Inês and crossbred SI/DO for the Student-Newman-Keuls test (SNK).

In relation to the corrected values of cells in stage 1 of the seminiferous epithelium cycle for the two groups studied, it was observed that the values of the cell types: spermatogonia, rounded spermatids and Sertoli cells were statistically similar. Nevertheless, there was difference between the primary spermatocyte in pre-leptotene/leptotene and pachytene, in which the number of these cell types was higher in Santa Inês (P < 0.05), according to Table 3 .

**Table 3:** Mean ± standard deviation of the spermatogenic and Sertoli cell population in stage 1 of the seminiferous epithelium cycle in Santa Inês (SI) and crossbred Santa Inês/Dorper (SI/DO).

Breeds	S	PL-L	PCT	RS	SC
SI-DO	8.49 ± 3.22^a^	13.18 ± 5.19^b^	23.48 ± 7.80^b^	84.73 ± 24.02^a^	9.30±4.18^a^
SI	8.46 ± 2.69^a^	15.36 ± 4.49^a^	27.42 ± 6.65^a^	84.82 ± 22.34^a^	9.78±3.50^a^

* Different letters in the same line expressed significant differences (P <0.05) between Santa Inês and crossbred SI/DO for the Student-Newman-Keuls test (SNK). Legend: S: Spermatogonia; PL/L: primary spermatocyte in pre-leptotene/leptotene; PCT: primary spermatocyte in pachytene; RS: rounded spermatid; CS: Sertoli cells.

For the yield of spermatogenic process, there are statistical differences between SantaInês and crossbred (Santa Inês/Dorper) for meiotic yield and the spermatogenesis yield, as shown in Table 4 . The mitotic coefficient and efficiency of Sertoli cells did not differ between the groups.

**Table 4:** Mean ± standard deviation of the spermatogenic yield in Santa Inês and crossbred Santa Inês/Dorper (SI/DO).

	Crossbred SI-DO	Santa Inês
Coefficient of mitoses (%)	0.58 ± 0.21^a^	0.61 ± 0.22^a^
Meiotic yield	3.98 ± 1.28^a^	3.31 ± 0.83^b^
General yeld	3.71 ± 1.02^a^	3.31 ± 1.20^b^
Sertoli Cell Efficiency	13.99 ± 11.45^a^	14.99 ± 13.99^a^

* Different letters in the same line expressed significant differences (P <0.05) between Santa Inês and crossbred SI/DO for the Student-Newman-Keuls test (SNK).

## Discussion

Studies have shown many factors that may influence the spermatogenic process, such as nutrition ( Viu *et al.,* 2006 ), temperature ( Marai *et al.,* 2008 ) and time of the year (rainy or dry; McManus *et al*., 2010 ; Santos *et al*., 2015 ). Recent studies also showed that the racial factor could lead to differences among the studied breeds (Andreusii *et al*., 2014).

In the present study, there was no variation between the groups regarding body weight, testicular weight and Gonadosomatic Index. The absence of difference for this parameter has the advantage of excluding variations between the groups in relation to the testicular size of the animals.

Although the variation in volume and proportion of testicular compartments denotes one of the main causes of differences observed in the spermatogenesis of the species ( França and Russell, 1998 ), in the racial comparison of the present study, the groups showed no difference in the density of these compartments.

Among the testicular compartments, the tubular compartments occupy 70-90% of the testicular parenchyma in mammals, as reported by França and Russell (1998) , and they influence on testis weight and sperm production (Aman, 1970). In the present study, the values obtained for the crossbred (77.02%) and Santa Inês (76.61%) are in accordance with the recommended standards. The values for these breeds are higher than those found in sheep with no racial pattern defined (71.04%; Martins *et al.,* 2008 ) and Morada Nova sheep (64.7%; Sousa, 2010 ), and lower than those reported in Suffolk sheep (83%; Wobrel *et al.*, 1995).

There is great variation in the tubular and interstitial compartment between the species ( França and Russell, 1998 ). The volume of the interstitial compartment in the seminiferous tubules showed values of 25.88% for crossbred and 25.94% for Santa Ines. Among the components of the interstitial compartment, the connective tissue was the most expressive, presenting almost equal values in both groups, as also found in non-defined breed sheep by Santos *et al*. (2015) . In the study performed by these authors, the volume of the interstitial compartment in the dry period of the year was 21.52%, which converges to the results presented. This percentage may also relate the samples collected during dry period of the year.

It is noteworthy the difference in the tubular diameter between crossbred (173.12 μm) and Santa Inês (185.71 μm), and also the height of the seminiferous epithelium (52.29 and 56.68 μm, respectively), evidencing higher values for Santa Inês. The height of the seminiferous epithelium was higher than that found in Santa Inês (46.67 and 48.26 μm; McManus *et al.,* 2010 ) and in non-defined breed sheep (44.92 and 50.06 μm; Santos *et al.,* 2015 ), and lower than those reported by Souza (2003) in Santa Inês (70.88 μm).

In relation to the tubular diameter, lower values than those found in the present study were reported in sheep with no racial pattern defined (164.2 μm; Martins *et al.,* 2008 ). They were also found in this same breed, in studies with samples collected in different periods of the year (dry and rainy season), with 152 and 171 μm, respectively ( Queiroz and Cardoso, 1989 ), and in Santa Inês, with 158.61 and 167.51 μm ( McManus *et al.,* 2010 ). Higher results have been reported in Santa Inês sheep, (207.12 μm) by Souza (2003) and other races as in Suffolk sheep (276.00 μm; Wrobel *et al,* 1995 ).

Although they are considered important indicators of spermatogenesis, it is possible to notice that intense variations occur in the height and diameter of the seminiferous tubules, even within the same racial group. According to Paula *et al*. (2002) , these variations are related to the structural elements of the tubules: the number of peritubular myoid cells that surround the *tunica propria*, the size and population of germ cells lineage and Sertoli cells, and the secreted fluid in the lumen of the seminiferous tubule. These factors may suffer variations into the same species, lineages or breeds, which explains these differences.

Regarding the frequencies of the stages that characterize the cycle of the seminiferous epithelium, there was no significant difference for any of the stages between the studied groups. However, it was observed that in the group of crossbred, the post-meiotic phase was faster (42.45%) than the one observed in the Santa Inês (45.10%). Therefore, it can be concluded that since in this group the final phase of spermatogenesis is processed faster, a greater amount of spermatozoa can be released in a shorter time.

There was no statistical difference for the spermatogonia population, nor there was any difference in the Sertoli cell population per cross-section of the seminiferous tubule, and the value of the latter was higher than in non-defined breedsheep (8.3; Martins *et al.,* 2008 ). In another study with Santa Inês sheep, McManus *et al.* (2010) found values of Sertoli cells in the rainy season close to those obtained in this work (10.44).

The number of Sertoli cells per testis is an indispensable factor in the determination of sperm production, and it has a correlation with the testicular weight. These cells have a fixed support capacity for the germ cells, so the more Sertoli cells exist in the testis, the more germ cells will be able to develop. The number of germ cells, mainly spermatids, that can be supported by a single Sertoli cell is an important indicator of the functional efficiency of the testis and, consequently, of sperm production ( França and Russell, 1998 ).

The crossbred presented lower numbers of primary spermatocytes in the pre-leptotene/leptotene and in pachytene, when compared to the Santa Inês breed, with values obtained within the limits found in sheep by Carrijo Júnior *et al*. (2008; 24 to 28). However, the number of rounded spermatids did not differ statistically for crossbred (84.73) when compared to Santa Inês (84.82). The evaluation of the spermatogenesis yield contributes to clarify better this process in the evaluation of the racial groups studied here.

The coefficient of mitoses establishes the degree of cell loss that occurred during this phase, and although it did not vary between breeds, it was below that found in the literature for sheep (1.78 and 1.90; Rodrigues *et al.,* 2016 ), without and with scrotal bipartition, respectively. However, this yield was close to that reported in goats, with a 50% of scrotal bipartition (1.1%) and to those without this morphological characteristic (1.11%; Machado Júnior *et al.*, 2012).

In the present study, the cellular losses occurred markedly, resulting in a lower number of spermatogonia. However, it is established that these losses are density-dependent, and limit the amount of cells that will enter in the meiosis process, regulating the cellular amount that may be supported by the Sertoli cells, in addition to the removal of defective cells ( De Rooij and Lok, 1987 ).

The amount of rounded spermatid observed in the two groups studied reflected the spermatogenic efficiency of these animals. The crossbred obtained a higher quantity of spermatid rounded per pachytene (3.98 ± 1.28) and a larger quantity of spermatid rounded per spematogonia (3.71 ± 1.02) when compared to Santa Inês, which obtained respectively (3.31 ± 0.83) and (3.31 ± 1.20) for the same parameters. The data correspond to the yield or meiotic index and general yield of spermatogenesis, evidencing a greater percentage of cellular loss for the Santa Inês group. In most domestic animals these losses are estimated between 60 and 90% ( França and Russell, 1998 ).

For some authors, cell loss occurs mainly by apoptosis in spermatogonia and spermatocytes, and there is relationship of these losses to factors such as photoperiod ( Young and Nelson, 2001 ), changes in FSH secretion ( Furuta *et al.,* 1994 ) and dietary restrictions ( Nelson *et al.,* 1992 ). In this case, it is possible that intrinsic, hormonal, animal related factors have caused interference, causing such losses at this point of spermatogenesis.

The crossbred presented better general spermatogenesis yield, an index that evaluates the spermatogenic process in a generalized way. Possibly the genetic characteristics acquired with crossbreeding have brought benefits to spermatogenesis in these animals through the combination of Santa Inês and Dorper characteristics. This fact may be verified in the research of Maia *et al.* (2011) , when comparing the same genotypes verified that crossbred has good semen and with a lower number of sperm defects, if examined in contrast with pure animals of the Dorper and Santa Inês breeds.

It is concluded there are differences in testicular morphology and spermatogenesis between Santa Inês sheep and crossbred Dorper/Santa Inês, indicating that the crossbreeding between the Santa Inês and Dorper animals allows a gain in the reproductive potential.
